# Supplementary material for: Neuron-specific isoform of PGC-1α regulates neuronal metabolism and brain aging
Source: Nat Commun. 2025 Feb 28;16:2053. doi: 10.1038/s41467-025-57363-y (PMC11871081; doi:10.1038/s41467-025-57363-y)
Supplement: Supplementary file 1 — Supplementary Information [file 41467_2025_57363_MOESM1_ESM.pdf]

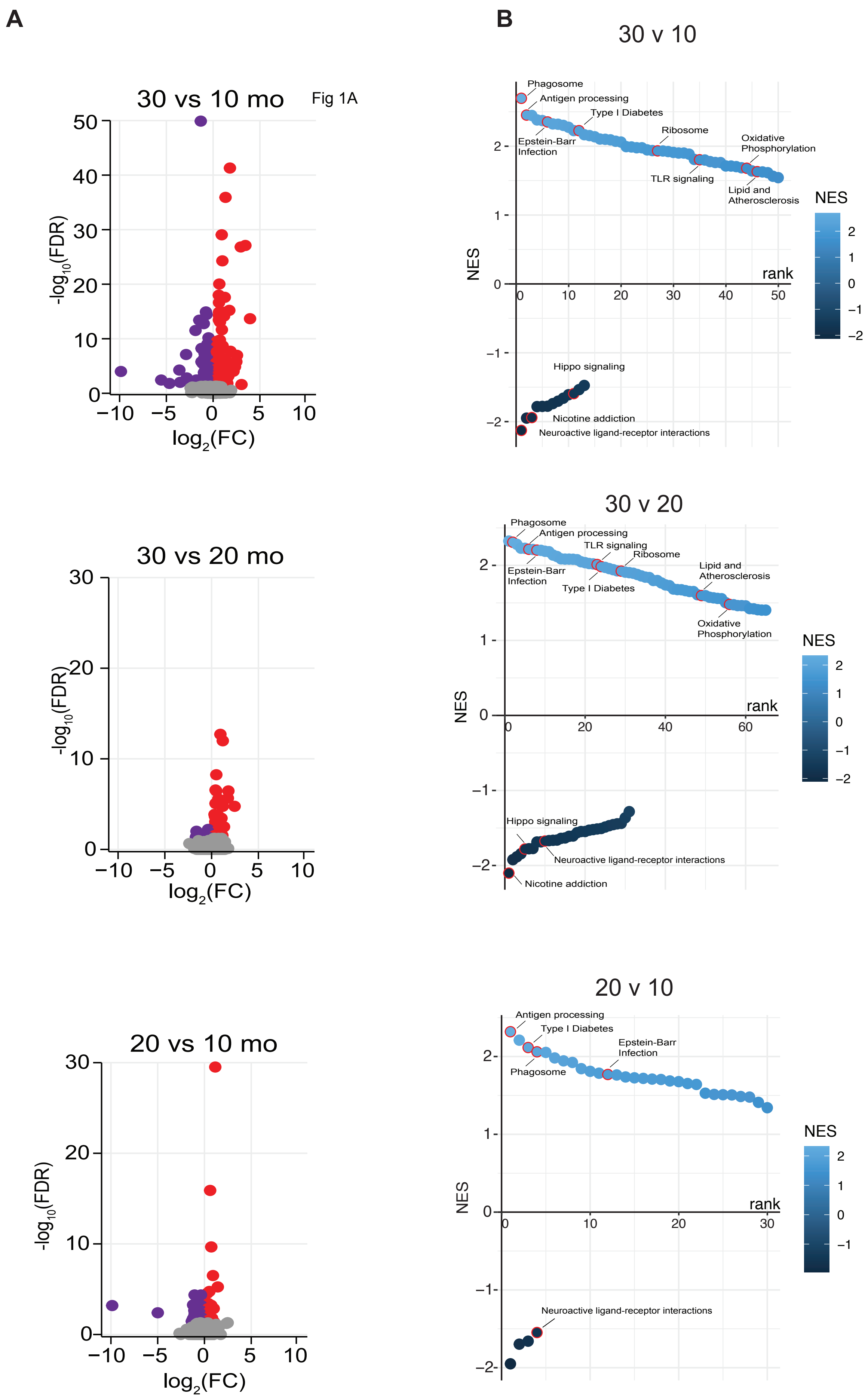
A
B
Fig 1A
30 vs 10 mo
-log10(FDR)
log2(FC)
30 vs 20 mo
20 vs 10 mo
30 v 10
Phagosome
Antigen processing
Type I Diabetes
Epstein-Barr Infection
Ribosome
Oxidative Phosphorylation
TLR signaling
Lipid and Atherosclerosis
NES
rank
Hippo signaling
Nicotine addiction
Neuroactive ligand-receptor interactions
30 v 20
Phagosome
Antigen processing
TLR signaling
Ribosome
Epstein-Barr Infection
Lipid and Atherosclerosis
Type I Diabetes
Oxidative Phosphorylation
NES
rank
Hippo signaling
Neuroactive ligand-receptor interactions
Nicotine addiction
20 v 10
Antigen processing
Type I Diabetes
Epstein-Barr Infection
Phagosome
NES
rank
Neuroactive ligand-receptor interactions

Figure S1: A) Volcano plots displaying transcripts quantified. Statistically significant transcripts are highlighted in red (upregulated) or purple (downregulated) in 30-month-old compared to 10-month-old male cortical tissue as shown in figure 1A. Equivalent volcano plots for 30-month-old compared to 20-month-old and 20-month-old compared to 10-month-old are shown. B) Rank order plot of enriched pathways detected by GSEA. Rank order plots for each of the three age comparsions are shown. Comparisons are 30-month vs 10-month, 30-month vs 20-month, and 20-month vs 10-month. Each plot is ranked by normalized enrichment score. n=5 mice per age group.

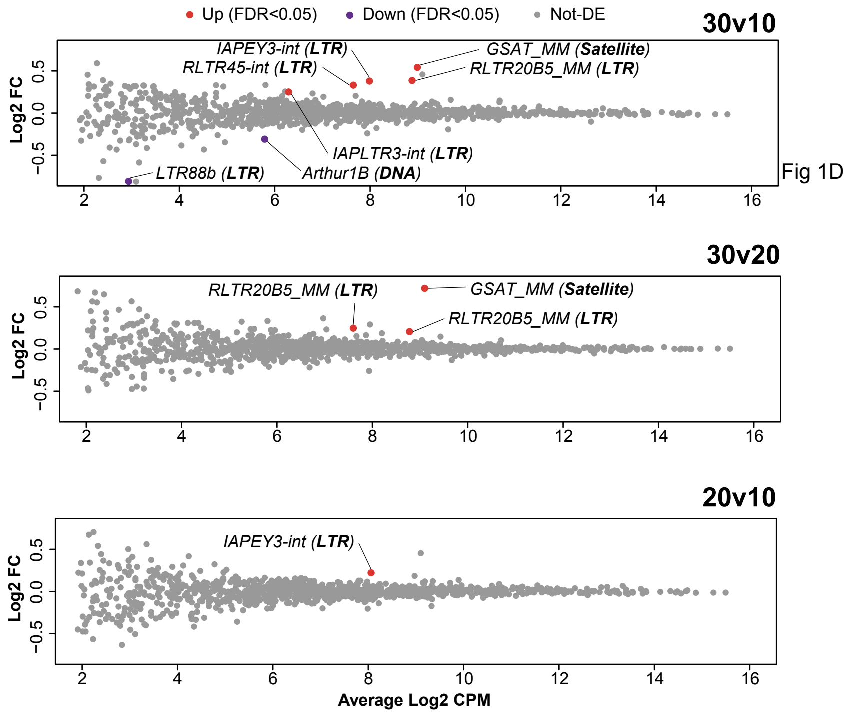


Figure S2: Mean-difference (MD) plots of transposable elements (TE) expression Log2 FC against the average Log2 count per-million (CPM) for the 30m/10m comparison (Fig 1D), 30m/20m comparison, and 20m/10m comparison.
TE callouts list the: “TE name (TE class).”

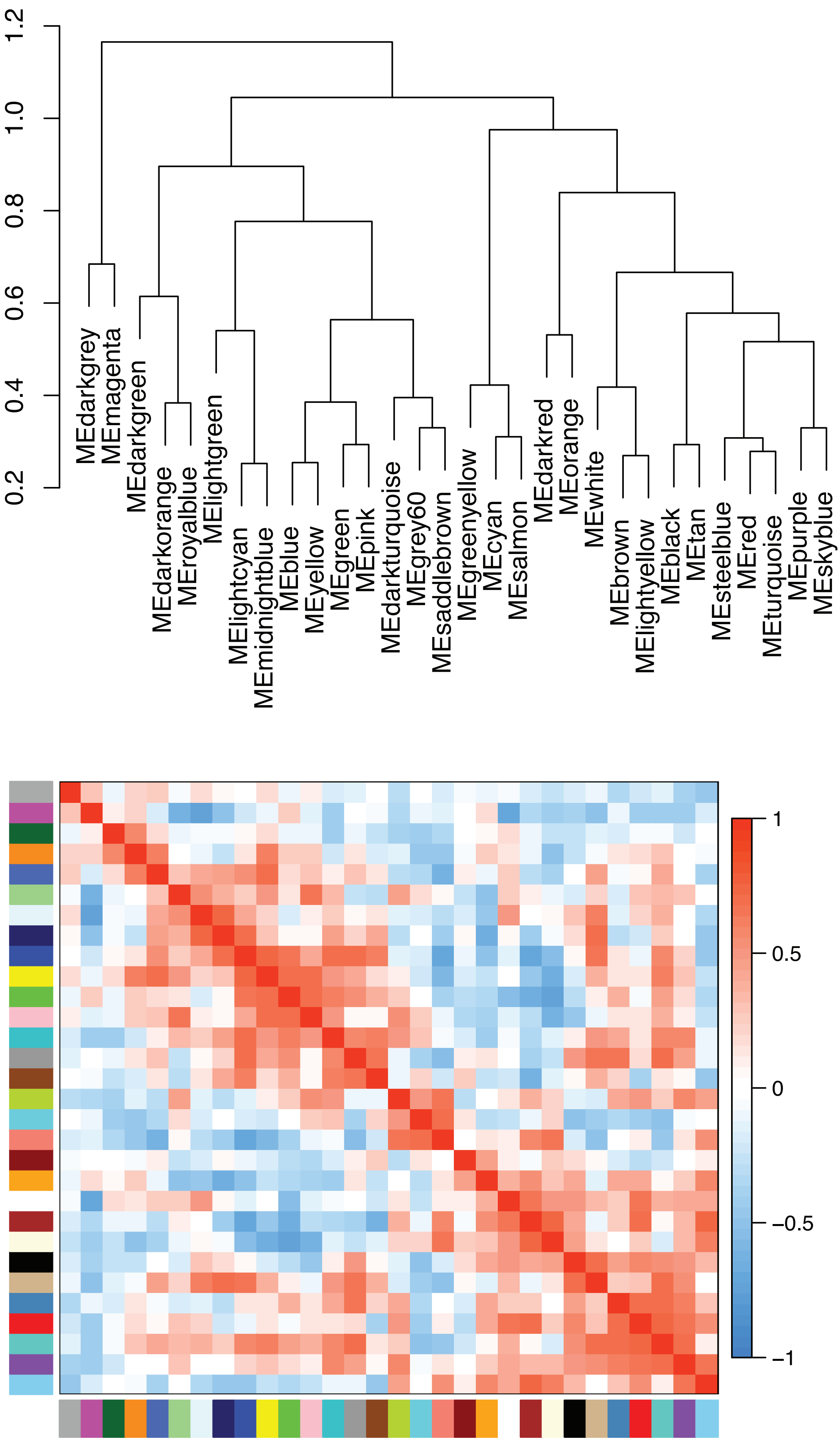


Figure S3: Dendrogram and heatmap displaying the correlation between the 30 modules detected by WGCNA. The heatmap shows the Pearson correlation from -1 to 1 between each of the modules graphed along the x and y axes.

**A**

| PGC-1α isoform | N-terminal amino acid sequence |
|---|---|
| PGC-1α1 | MAWDMCSQDSVWSDIE - Exon2 |
| PGC-1α4 | MLGLSSMDSILK - Exon2 |
| PGC-1α B1E2 | MDEGYF - Exon2 |

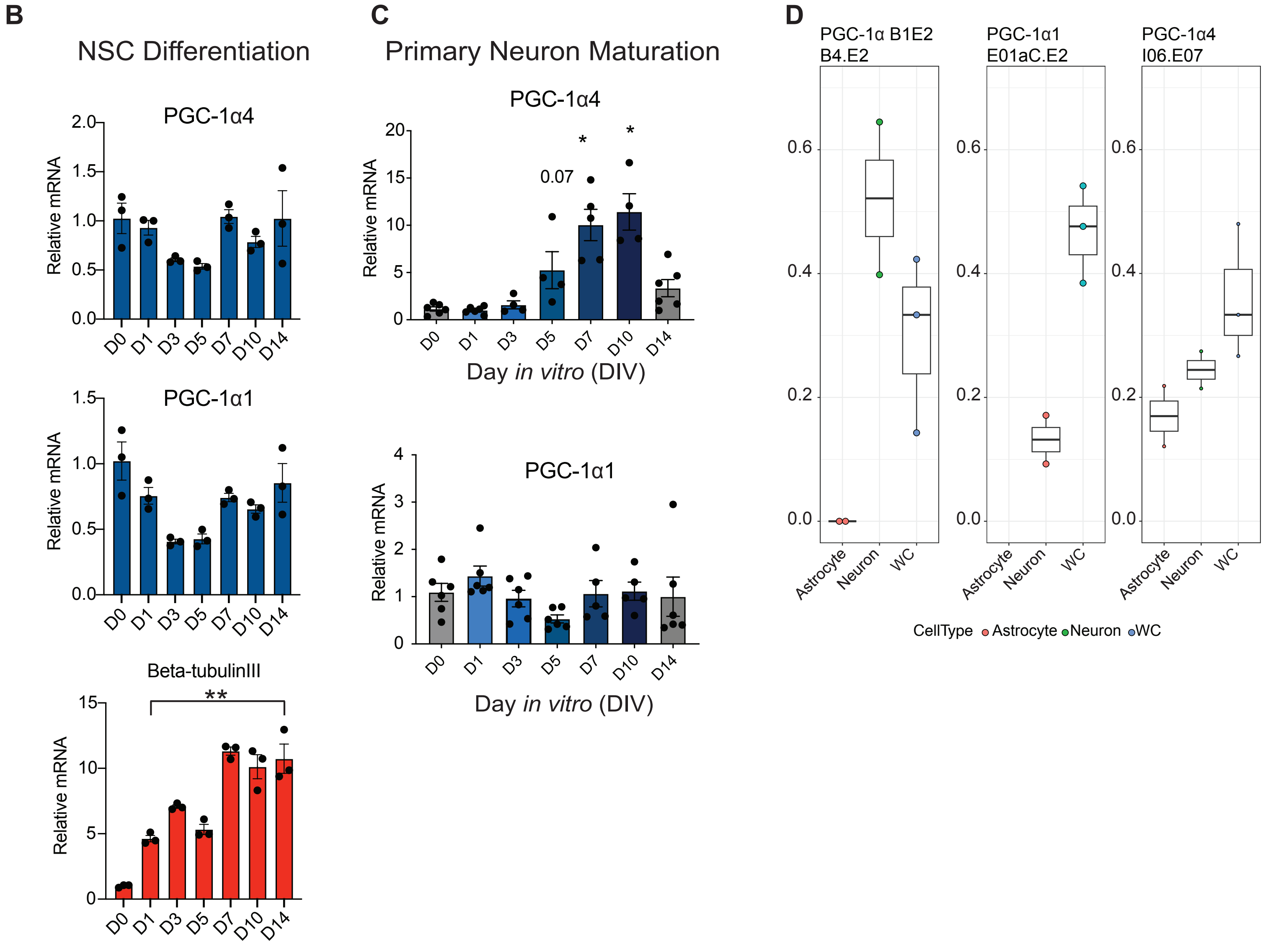


Figure S4: A) Table displaying the N-terminal amino acid sequences for the three major isoforms of PGC-1α expressed in neurons.
B) RT-qPCR detection of the canonical PGC-1α1, alternative PGC-1α4 isoform, and neuronal marker beta-tubulin III during neural stem cell (NSC) differentiation (n=3 cultures/ differentiation day). Significance was determined by one-way ANOVA. D1: p=0.0033, D3:p<0.0001, D5: p=0.0007, D7:p<0.0001, D10:p<0.0001, D14:p<0.0001. C) Detection of PGC-1α1 and PGC-1α4 during maturation of primary cortical neurons (PGC1α4: DIV0, 1, 14: n=6, DIV3, 5, 10: n=4; DIV7: n=5; PGC1α1: DIV0, 1, 3, 5, 14: n=6; DIV7, 10: n=5 cortical cultures/ maturation day). D) Boxplots of normalized exon junction read counts for the brain (brain exon 4 – canonical exon 2), α1 (canonical exon 1 – canonical exon 2) and α4 (intron 6 – canonical exon 7) Ppargc1a isoforms from mouse brain RNA-seq (n=2 astrocytes, n=2 neurons, n=3 whole cortex).
Data shown as mean +/- SEM (B,C) or Boxplot represents the median, 25th and 75th percentiles, and whiskers extend to min and max unless +/- 1.5 IQR (D).

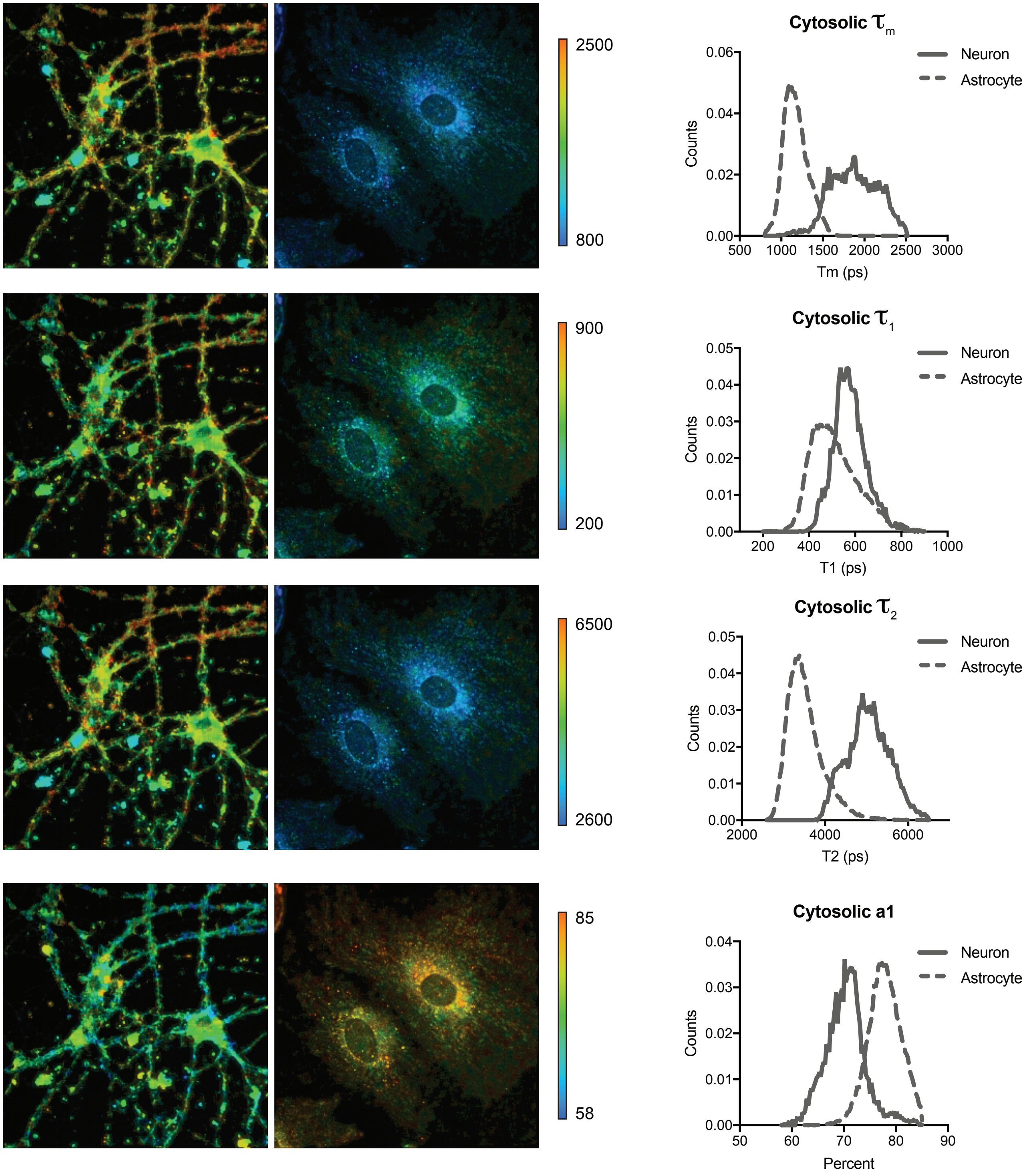


Figure S5: Representative images (left)and distributions (right) of NAD(P)H fluorescence lifetime images of primary neurons and primary astrocytes. Representative images are artificially colored to show differences in mean fluorescence lifetime, the short component of the decay curve (T1), the long component of the decay curve(T2), or the relative contribution of free NAD(P)H (a1) to the mean fluorescence lifetime (n=10 neurons, n=12 astrocytes).

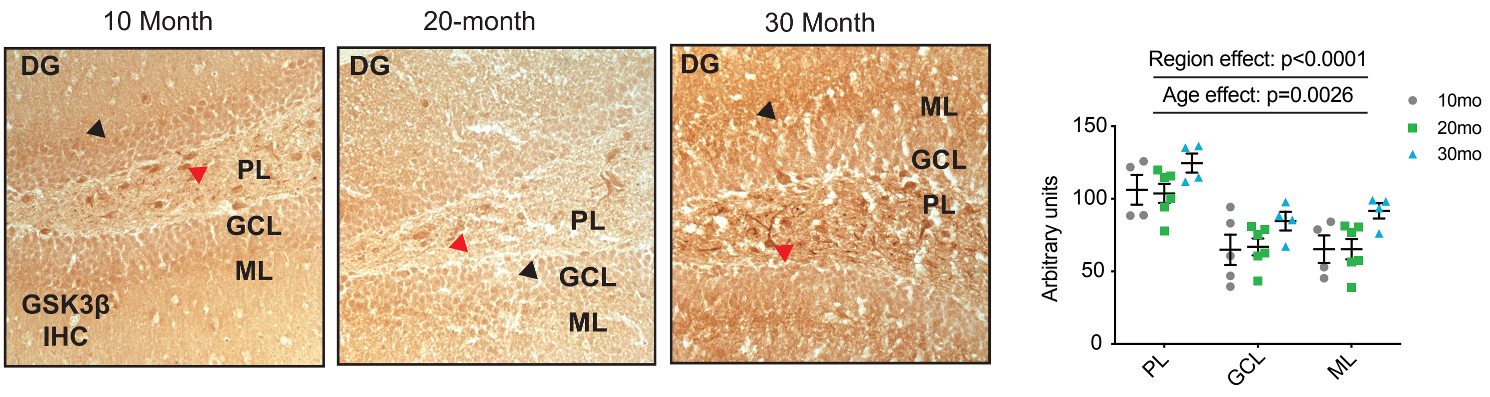


Figure S6: Immunohistochemical detection of GSK3β in the indicated hippocampal regions for 10-month-old, 20-month-old, and 30-month-old mice (n=4 30-month-old mice, n=6 10- and 20-month-old mice). Statistical significance was determined by orinary 2-way ANOVA. Data shown as mean +/- SEM.

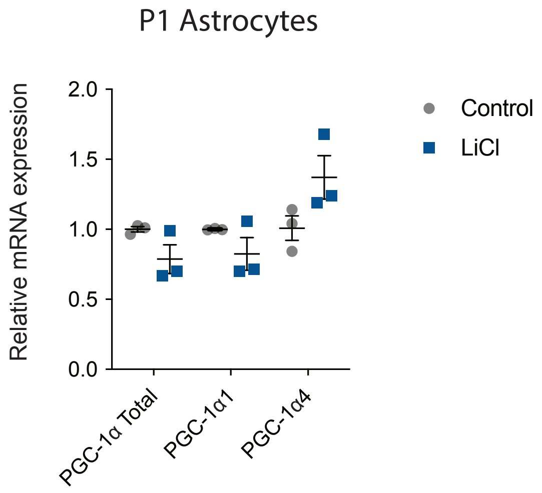


Figure S7: RT-qPCR detection of PGC-1α transcripts in control and LiCl-treated P1 astrocytes (n=3 astrocyte cultures). Statistical significance was determined by unpaired two-tailed Student's t-test for each PGC-1α transcript. Data shown as mean +/- SEM.

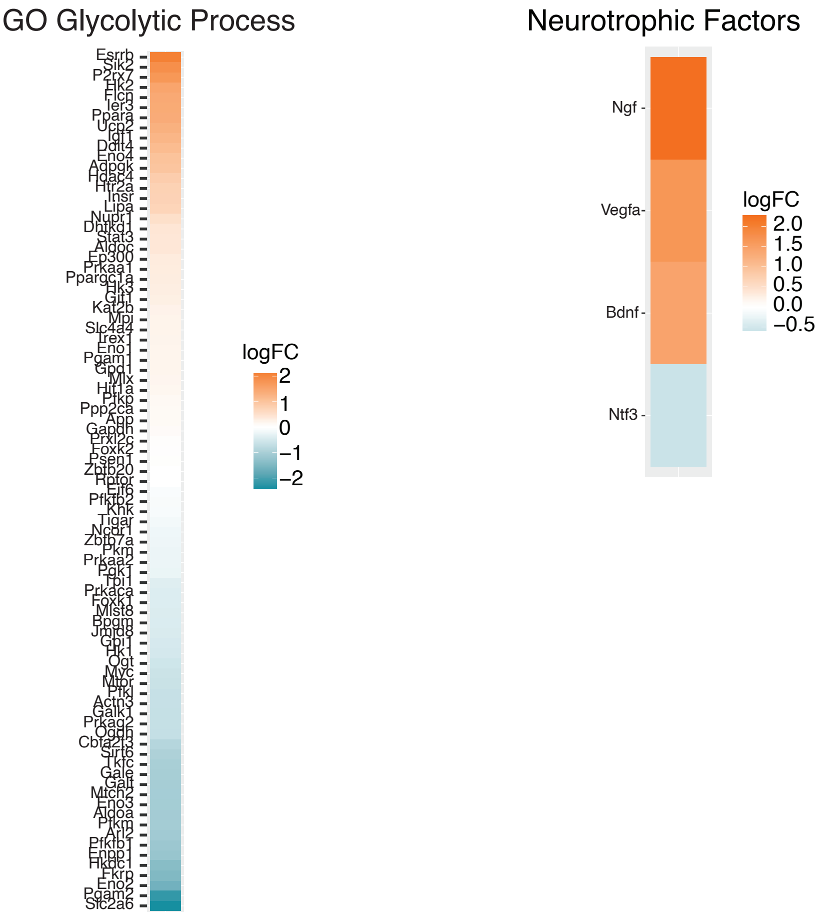


Figure S8: Heatmaps of the genes in the “Glycolysis” GO term and significantly changing neurotrophic factors in primary neurons treated with LiCl.

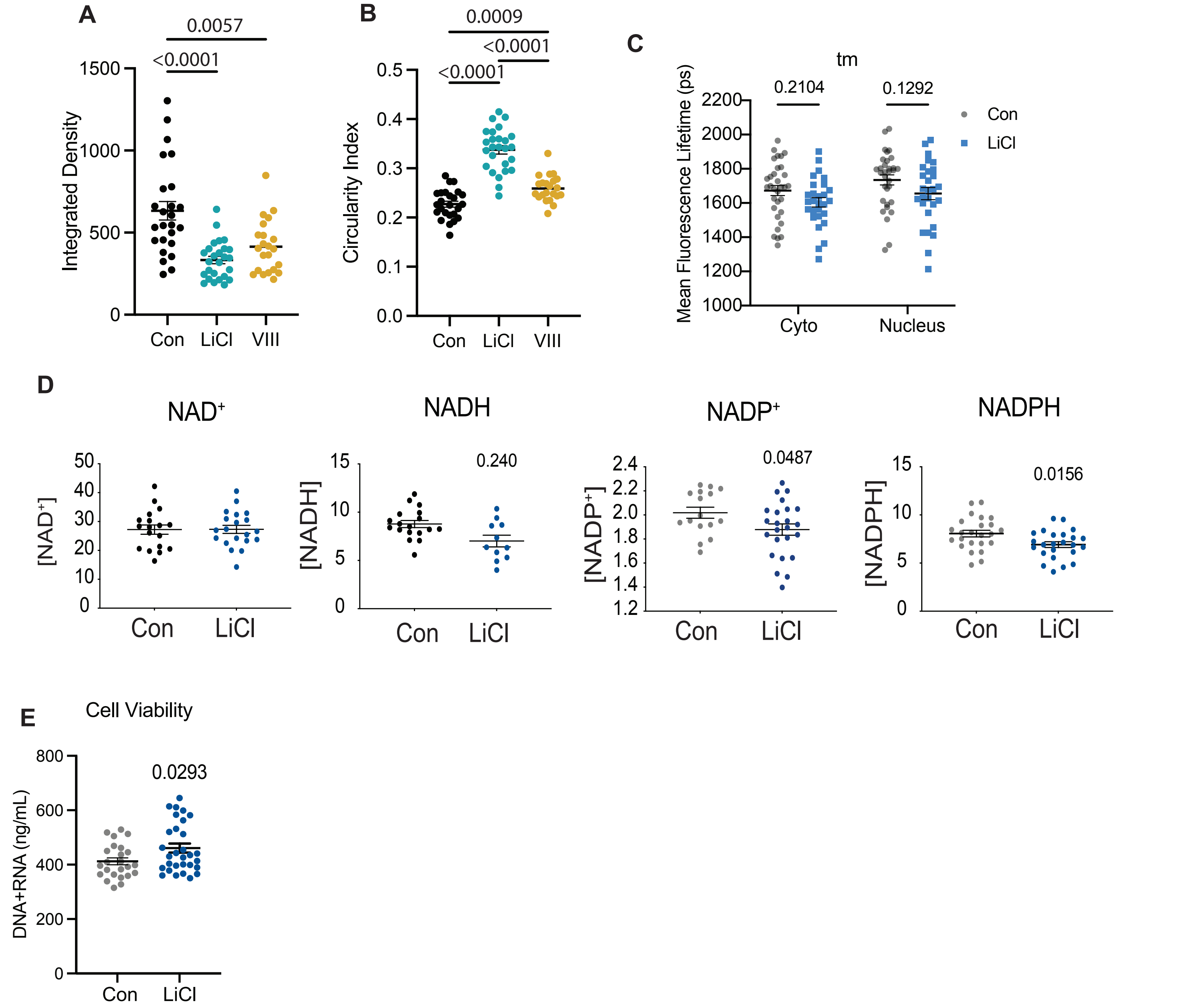


Figure S9: A-B) Mitochondrial morphology of TOMM20 labeled mitochondria (Fig 5G). Analysis was done in ImageJ to detect integrated density (product of mean intensity and mitochondrial area) (n=25 Control, n=26 LiCl, n=22 VIII) (A), and circularity (n=25 Control, n=26 LiCl, n=22 VIII) (B). C) Average mean fluorescence lifetime of control or LiCl-treated primary neurons. Each dot represents the mean Tm from one cell (n=32 control, n=28 LiCl). D) NAD, NADH, NADP, and NADPH concentrations determined by biochemical assay. E) Cell viability of primary neurons treated with LiCl for 24 hours measured by CyQuant assay (n=24 control, n=30 LiCl). Statstical signficance determined by Brown-Forsythe and Welch one-way ANOVA  with Dunnett's test (A,B), two-way ANOVA with Sidak's test (C), or two-tailed unpaired student's t-test (D,E). Data shown as mean +/- SEM.

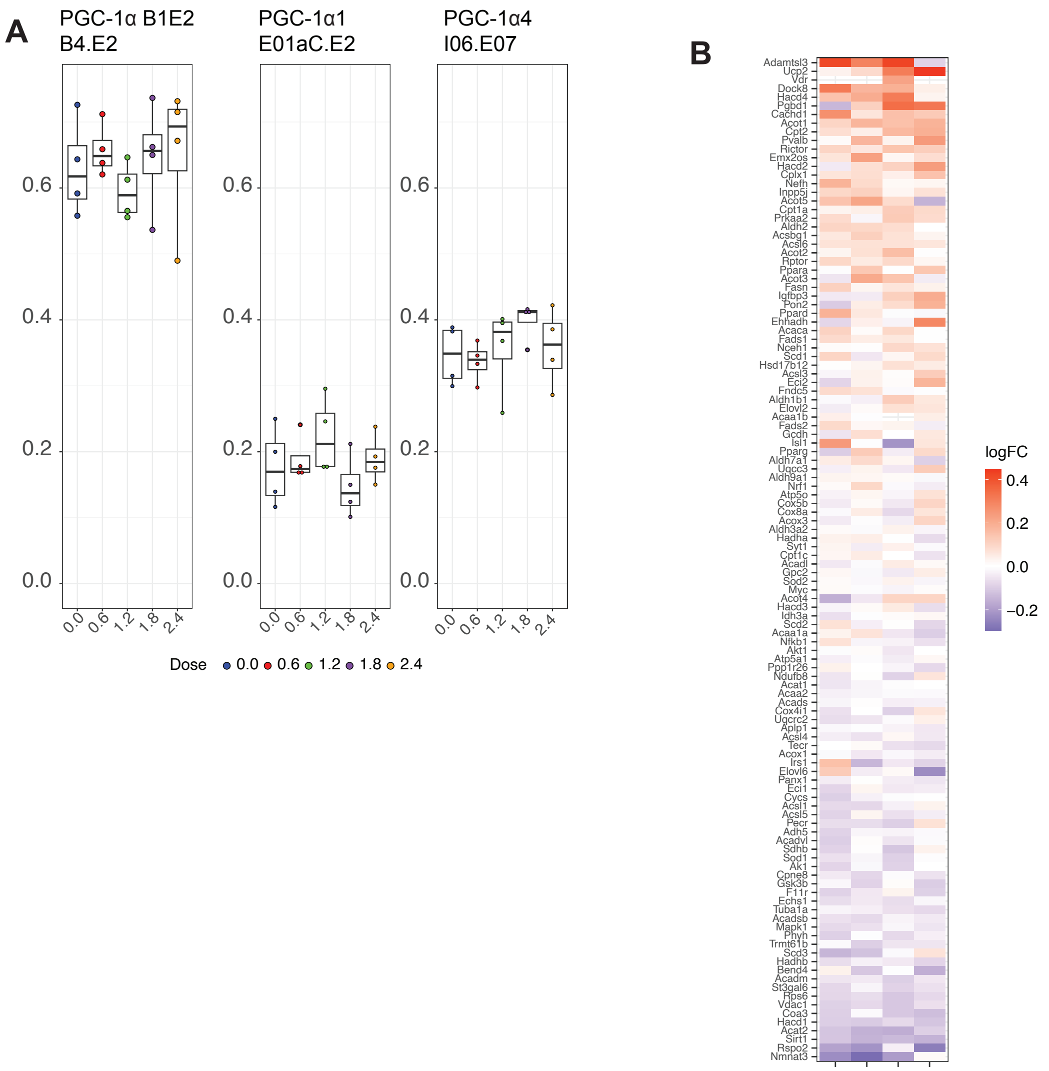


Figure S10: A) Boxplots of normalized exon junction read counts for the brain (brain exon 4 – canonical exon 2), α1 (canonical exon 1 – canonical exon 2) and α4 (intron 6 – canonical exon 7) Ppargc1a isoforms from mouse brain RNA-seq (n=4 mice) across the different LiCO3 diet dosages. B) Heatmaps of the PGC-1α-associated genes term detected across each of the four doses of $Li_2CO_3$.

Uncropped western blots associated with Figure 4

Fig 4C - pGSK3β (S9)

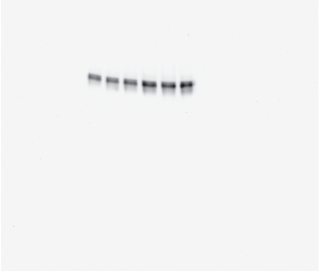

Fig 4C - GSK3β

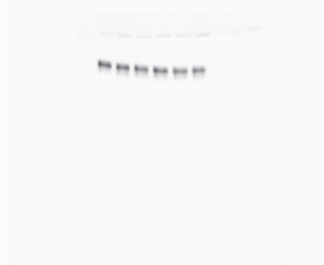

Fig 4C - pCREB (S133)

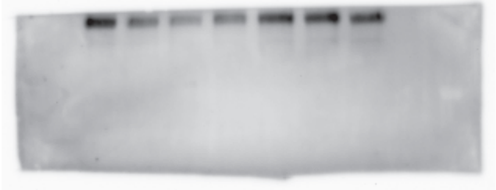

Fig 4C - CREB

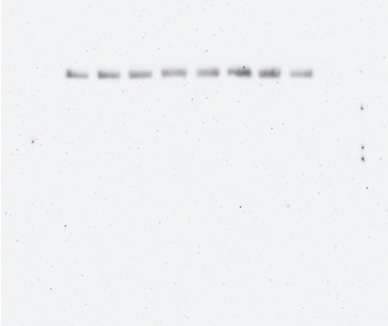

Fig 4C - pAMPK (T172)

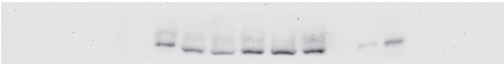

Fig 4C - AMPK

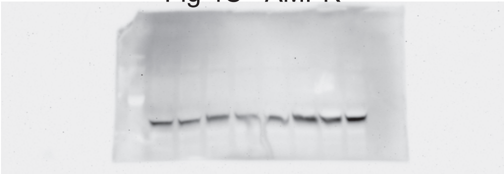

Uncropped western blots associated with Figure 6

Fig 6A - pIRS (S636)

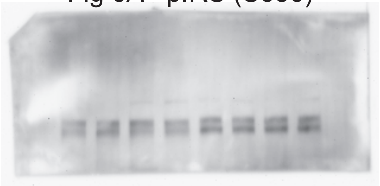

Fig 6A - IRS

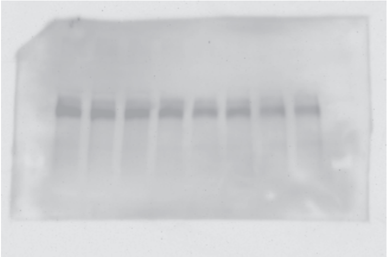

Fig 6A - pAKT (S308)

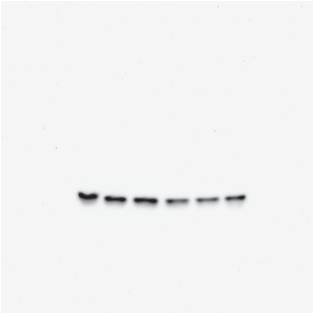

Fig 6A - AKT

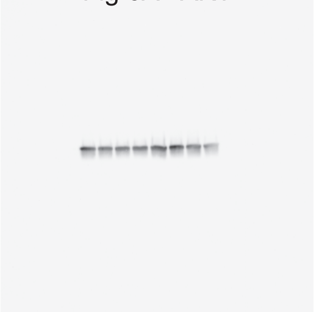

Fig 6A - pS6 (S240/244)



Fig 6A - S6



Fig 6A - pERK1/2 (T202/ Y204)

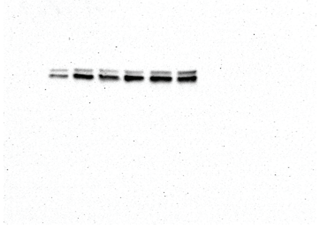

Fig 6A - ERK 1/2

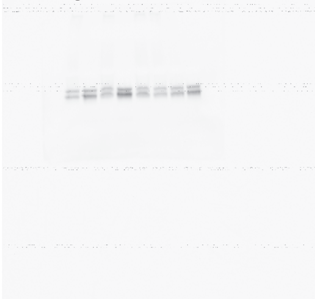

Figure S11: Raw western blot images for figures 4 and 6.
