# Supplementary material for: Neuron-specific isoform of PGC-1α regulates neuronal metabolism and brain aging
Source: Nat Commun. 2025 Feb 28;16:2053. doi: 10.1038/s41467-025-57363-y (PMC11871081; doi:10.1038/s41467-025-57363-y)
Supplement: Supplementary file 2 — Description of Additional Supplementary Files [file 41467_2025_57363_MOESM2_ESM.pdf]

Description of Additional Supplementary Files

Supplementary Data 1: Differentially expressed genes and enriched KEGG pathways detected by GSEA of aged mouse brains. Enriched pathways were determined using an FDR<0.05.

Supplementary Data 2: Modules produced by WGCNA of aged mouse brains.

Supplementary Data 3: Transposable elements detected in the aging brain RNA-Seq dataset. Significance determined by FDR< 0.05.

Supplementary Data 4: Differentially expressed proteins and significantly enriched KEGG pathways detected by overrepresentation analysis in aged mouse brains. Statistically significant proteins were determined by Tukey's HSD test.

Supplementary Data 5: RT-qPCR primer sequences for PGC-1a transcript variants.

Supplementary Data 6: Differentially expressed genes and significantly enriched pathways detected by gene set enrichment analysis in LiCl-treated neurons. DE genes were determined as FDR<0.05. Enriched pathways were determined using a p.adjust<0.05. The adjustment method was FDR.

Supplementary Data 7: Differentially expressed genes and significantly enriched pathways detected in brains of LiCO3-fed mice. Significant pathways were determined by an FDR <0.05.
