# Supplementary material for: Neuron-specific isoform of PGC-1α regulates neuronal metabolism and brain aging
Source: Nat Commun. 2025 Feb 28;16:2053. doi: 10.1038/s41467-025-57363-y (PMC11871081; doi:10.1038/s41467-025-57363-y)
Supplement: Supplementary file 10 — Reporting Summary [file 41467_2025_57363_MOESM10_ESM.pdf]

nature portfolio

Corresponding author(s): *Double-blind peer review submissions: write DBPR and your manuscript number here instead of author names.*

Last updated by author(s): Feb 5, 2025

Reporting Summary

Nature Portfolio wishes to improve the reproducibility of the work that we publish. This form provides structure for consistency and transparency in reporting. For further information on Nature Portfolio policies, see our Editorial Policies and the Editorial Policy Checklist.

Statistics

For all statistical analyses, confirm that the following items are present in the figure legend, table legend, main text, or Methods section.

n/a	Confirmed	
☐	☒	The exact sample size (*n*) for each experimental group/condition, given as a discrete number and unit of measurement
☐	☒	A statement on whether measurements were taken from distinct samples or whether the same sample was measured repeatedly
☐	☒	The statistical test(s) used AND whether they are one- or two-sided<br>*Only common tests should be described solely by name; describe more complex techniques in the Methods section.*
☒	☐	A description of all covariates tested
☐	☒	A description of any assumptions or corrections, such as tests of normality and adjustment for multiple comparisons
☐	☒	A full description of the statistical parameters including central tendency (e.g. means) or other basic estimates (e.g. regression coefficient) AND variation (e.g. standard deviation) or associated estimates of uncertainty (e.g. confidence intervals)
☒	☐	For null hypothesis testing, the test statistic (e.g. *F*, *t*, *r*) with confidence intervals, effect sizes, degrees of freedom and *P* value noted<br>*Give P values as exact values whenever suitable.*
☐	☒	For Bayesian analysis, information on the choice of priors and Markov chain Monte Carlo settings
☐	☒	For hierarchical and complex designs, identification of the appropriate level for tests and full reporting of outcomes
☒	☐	Estimates of effect sizes (e.g. Cohen's *d*, Pearson's *r*), indicating how they were calculated

Our web collection on statistics for biologists contains articles on many of the points above.

Software and code

Policy information about availability of computer code

Data collection	RNA expression data was analyzed from one publically available dataset GSE52564. The GEO accession id for the new RNA-seq data gathered for this study is GSE246478. Proteomics data is available on Mendeley data (DOI: 10.17632/vcv2nyp7s3.1).
Data analysis	RNA-Sequencing: Sequencing reads were trimmed to remove sequencing adaptors and low-quality bases, aligned to the mm10 reference genome using the STAR aligner, and alignments used as input to RSEM for quantification. Differential gene expression analysis was performed via EdgeR generalized linear model (GLM) method. GSEA analysis was conducted using standard R packages (fgsea).<br>Proteomics: Peptide and protein identifications were accepted under strict 1% FDR cut offs, at least two peptide spectrum matches per protein and with high confidence XCorr thresholds of 1.9 for z=2 and 2.3 for z=3. Strict principles of parsimony were applied for protein grouping. Chromatograms were aligned for feature mapping and ion intensities were used for precursor ion quantification using unique and razor peptides. Normalization was performed on total peptide amount and scaling on all average. Protein abundance was determined from summed peptide abundances and ANOVA (individual proteins) was executed for hypothesis testing.

For manuscripts utilizing custom algorithms or software that are central to the research but not yet described in published literature, software must be made available to editors and reviewers. We strongly encourage code deposition in a community repository (e.g. GitHub). See the Nature Portfolio guidelines for submitting code & software for further information.

Data

Policy information about availability of data

All manuscripts must include a data availability statement. This statement should provide the following information, where applicable:

- Accession codes, unique identifiers, or web links for publicly available datasets
- A description of any restrictions on data availability
- For clinical datasets or third party data, please ensure that the statement adheres to our policy

The GEO accession id for the new RNA-seq data gathered for this study is GSE246478. Proteomics data is available on Mendeley data (DOI: 10.17632/vcv2nyp7s3.1). All other datasets are available from the corresponding author upon reasonable request.

Human research participants

Policy information about studies involving human research participants and Sex and Gender in Research.

Reporting on sex and gender	*Use the terms sex (biological attribute) and gender (shaped by social and cultural circumstances) carefully in order to avoid confusing both terms. Indicate if findings apply to only one sex or gender; describe whether sex and gender were considered in study design whether sex and/or gender was determined based on self-reporting or assigned and methods used. Provide in the source data disaggregated sex and gender data where this information has been collected, and consent has been obtained for sharing of individual-level data; provide overall numbers in this Reporting Summary. Please state if this information has not been collected. Report sex- and gender-based analyses where performed, justify reasons for lack of sex- and gender-based analysis.*
Population characteristics	*Describe the covariate-relevant population characteristics of the human research participants (e.g. age, genotypic information, past and current diagnosis and treatment categories). If you filled out the behavioural & social sciences study design questions and have nothing to add here, write "See above."*
Recruitment	*Describe how participants were recruited. Outline any potential self-selection bias or other biases that may be present and how these are likely to impact results.*
Ethics oversight	*Identify the organization(s) that approved the study protocol.*

Note that full information on the approval of the study protocol must also be provided in the manuscript.

Field-specific reporting

Please select the one below that is the best fit for your research. If you are not sure, read the appropriate sections before making your selection.

[x] Life sciences [ ] Behavioural & social sciences [ ] Ecological, evolutionary & environmental sciences

For a reference copy of the document with all sections, see nature.com/documents/nr-reporting-summary-flat.pdf

Life sciences study design

All studies must disclose on these points even when the disclosure is negative.

Sample size	*Describe how sample size was determined, detailing any statistical methods used to predetermine sample size OR if no sample-size calculation was performed, describe how sample sizes were chosen and provide a rationale for why these sample sizes are sufficient.*
Data exclusions	Outliers were identified by ROUT using a threshold of Q = 0.05.
Replication	All data capture experiments were conducted in samples derived from a minimum of four cultures (each culture was composed of 2 pooled cortices). Each image-based data capture experiment involved at least five individual cells per culture per treatment.
Randomization	For each drug treatment in cell culture, samples from the same animal were used in each treatment.
Blinding	*Describe whether the investigators were blinded to group allocation during data collection and/or analysis. If blinding was not possible, describe why OR explain why blinding was not relevant to your study.*

Reporting for specific materials, systems and methods

We require information from authors about some types of materials, experimental systems and methods used in many studies. Here, indicate whether each material, system or method listed is relevant to your study. If you are not sure if a list item applies to your research, read the appropriate section before selecting a response.

Materials & experimental systems

n/a	Involved in the study
☐	☒ Antibodies
☒	☐ Eukaryotic cell lines
☒	☐ Palaeontology and archaeology
☐	☒ Animals and other organisms
☒	☐ Clinical data
☒	☐ Dual use research of concern

Methods

n/a	Involved in the study
☒	☐ ChIP-seq
☒	☐ Flow cytometry
☒	☐ MRI-based neuroimaging

Antibodies

Antibodies used

Antibodies used for western blotting were: pGSK3b(S9) (#9336; Cell Signaling Technologies), GSK3b (9315S; Cell Signaling Technologies), pCREB (S133) (ab32096; Abcam), CREB (#9104; Cell Signaling Technologies), pAMPK (T172) (#2535S; Cell Signaling Technologies), AMPK (#2532S; Cell Signaling Technologies), pIRS-1 (S636) (#2388S; Cell Signaling Technologies), IRS-1 (#2382S; Cell Signaling Technologies), pAKT (T308) (#13038S; Cell Signaling Technologies), AKT (#4691; Cell Signaling Technologies), pS6 (S240/244) (#2215S; Cell Signaling Technologies), S6 (#2217S; Cell Signaling Technologies), pERK1/2 (T202/ Y204) (#4370S; Cell Signaling Technologies), ERK1/2 (#4695S; Cell Signaling Technologies).

rimary antibodies used for immunofluorescence were anti-a-Tubulin primary antibody (1:200) (Sigma-Aldrich #T6199) and Tomm20 (1:84.2) (Abcam ab56783).

Validation

All antibodies used have been validated for specificity by their manufacturers.

Animals and other research organisms

Policy information about studies involving animals; ARRIVE guidelines recommended for reporting animal research, and Sex and Gender in Research

Laboratory animals

Primary cortical neurons were isolated from the brains of P0 C57BL6J mice.

Wild animals

Provide details on animals observed in or captured in the field; report species and age where possible. Describe how animals were caught and transported and what happened to captive animals after the study (if killed, explain why and describe method; if released, say where and when) OR state that the study did not involve wild animals.

Reporting on sex

For all brain data (aging brain RNA-seq, aging brain proteomics, and lithium diet brain) all mice used were male mice.

Field-collected samples

For laboratory work with field-collected samples, describe all relevant parameters such as housing, maintenance, temperature, photoperiod and end-of-experiment protocol OR state that the study did not involve samples collected from the field.

Ethics oversight

All animal protocols were approved by the University of Wisconsin Institutional Animal Care and Use Committee (MV1774 – aging; MV2579 – Lithium diet) or the Institutional Animal Care and Use Committee at the William S. Middleton Memorial Veterans Hospital (RA0006 – primary cell isolation).

Note that full information on the approval of the study protocol must also be provided in the manuscript.
